# Tirzepatide and SGLT2 Inhibitors for Heart Failure With Preserved Ejection Fraction and Obesity: Entering a New Cardiometabolic Therapeutic Era

**DOI:** 10.1002/edm2.70298

**Published:** 2026-07-30

**Authors:** Zakariye Isak Mohamed, Abdinasir Adam Shidane, Abdullahi Mohamud Abdiasis, Mohamed Hassan Hussein, Ayaanle Osman Ibrahim, Anas Ali Abdi, Kowthar Ismail Hashi, Mohamed Abdullahi Mohamud, Ali Jimale Mohamed

**Affiliations:** ^1^ Faculty of Medicine and Surgery Somali National University Mogadishu Somalia; ^2^ Faculty of Medicine Al‐Azhar University Cairo Egypt; ^3^ Department of Cardiology Jazeera Specialist Hospital Mogadishu Somalia; ^4^ Department of Pharmacology, Faculty of Medicine and Surgery Somali National University Mogadishu Somalia

**Keywords:** dual GIP/GLP‐1 receptor agonist, heart failure with preserved ejection fraction, pharmacological synergy, SGLT2 inhibitors, tirzepatide

## Abstract

**Background:**

Heart failure with preserved ejection fraction (HFpEF) accounts for over half of all heart failure hospitalizations, with obesity increasingly recognized as a central driver through expansion of epicardial adipose tissue, chronic low‐grade inflammation and obesity‐related haemodynamic overload culminating in ventricular hypertrophy.

**Methods:**

We synthesized high‐impact evidence from pivotal randomized trials and mechanistic studies published between 2015 and 2026, focusing on cardiometabolic outcomes, adipose tissue remodelling and haemodynamic effects of both drug classes.

**Results:**

Tirzepatide produced substantial weight loss (> 20%), significant reductions in epicardial and paracardiac adipose tissue and a 19.5‐point improvement in Kansas City Cardiomyopathy Questionnaire scores in the SUMMIT trial. SGLT2i, exemplified by empagliflozin, demonstrated foundational benefit through natriuresis, improved myocardial energetics and reduced heart failure hospitalization independent of diabetes status, with additional renal‐protective effects. Because SUMMIT and EMPEROR‐Preserved differed in design and populations, direct comparison between agents is not statistically valid; each should be assessed on its own evidence base.

**Conclusion:**

Tirzepatide and SGLT2i act through distinct, physiologically complementary pathways converging on the cardio‐renal‐metabolic axis. While their combined use is mechanistically promising, it has not yet been tested in a dedicated randomized controlled trial, and prospective studies are needed to establish safety and long‐term efficacy in obesity‐related HFpEF.

## Graphical abstract

Tirzepatide and SGLT2 inhibitors act through distinct but complementary mechanisms in obesity‐related HFpEF: tirzepatide targets adiposity, inflammation, and insulin resistance, while SGLT2 inhibitors improve decongestion, myocardial energetics, and cardiac remodeling. Each drug class carries a distinct adverse‐effect profile, and their concurrent use may compound risks. As no dedicated randomized trial has evaluated this combination in HFpEF, combined use remains a physiologically rational but unproven strategy.

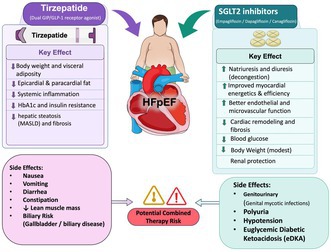

**Abbreviations**
ACEI: Angiotensin Converting Enzyme
ANP: Atrial Natriuretic Peptide
ARBS: Angiotensin Receptor Blockers
ARNI: Angiotensin Receptor And Neprilysin Inhibitor
AST: Aspartate Aminotransferase
BP: Blood Pressure
BV: Blood Volume
CRP: C‐Reactive Protein
EAT: Epicardial Adipose Tissue
eGFR: Estimated Glomerular Filtration Rate
FFAs: Free Fatty Acids
GIP: Glucose‐Dependent Insulinotropic Peptide
GLP‐1: Glucagon‐Like Peptide 1
HDL: High‐Density Lipoprotein
HF: Heart Rate
HFE: Heart Failure Exacerbation
HFPEF: Heart Failure With Preserved Ejection Fraction
HFrEF: Heart Failure With Reduced Ejection Fraction
KCCQ: Kanase City Cardiomyopathy Questnaire
LDL: Low‐Density Lipoprotein
LDL: Low‐Density Lipoprotein Cholesterol
LV: Left Ventricle
LVEDV: Left Ventricle End Diastolic Volume
LVEF: Left Ventricular Ejection Fraction
MACE: Major Adverse Cardiovascular Events
MASH: Metabolic Dysfunction‐Associated Steatohepatitis
MASLD: Metabolic Dysfunction‐Associated Steatotic Liver Disease
MRA: Mineralocorticoid Receptor Antagonist
NAFLD: Non‐Alcoholic Fatty Liver Disease
PAT: Paracardial Adipose Tissue
PV: Plasma Volume
SGLT2i: Sodium‐Glucose Transporter‐2 inhibitor
T2D: Type Two Diabetes
TG: Triglyceride
WHO: World Health Organization

## 1 Introduction

Heart failure is one of the leading causes of death in cardiovascular disease; over half of hospitalized patients with heart failure (HF) have heart failure with preserved ejection fraction (HFpEF) [1, 2, 3]. Various reports highlighted the contribution of type 2 diabetes and obesity in initial developmental heart failure, especially HFpEF, and its worsening in patients with an established diagnosis of HFpEF [4]. However, obesity, and especially upper body (central) obesity, correlates not only with a heightened risk for HFpEF but also for other cardiovascular sequelae such as dyslipidaemia, hypertension and type 2 diabetes [2]. Additionally, obesity is linked to elevated cardiac output and blood volume, which cause dilatation of the left ventricle and eventual hypertrophy due to increased wall stress [2, 5]. Obese HFpEF is also associated with increased epicardial adipose tissue (EAT) [2, 6]. EAT may contribute to HFpEF via the action of locally secreted proinflammatory adipocytokines, which may directly cause cardiac muscle remodelling and dysfunction, compress the heart and impair filling and reduce natriuretic peptide secretion due to decreased chamber distention [2, 6].

Previously, pharmacological management of heart failure involved agents such as angiotensin‐converting enzyme (ACE) inhibitors, beta‐blockers, angiotensin receptor blockers (ARBs) and diuretics [7]. Recent clinical trials provide evidence that tirzepatide, a dual GLP‐1/GIP receptor agonist and incretin mimetic, offers significant therapeutic benefit in the management of heart failure with preserved ejection fraction (HFpEF) [8]. Tirzepatide has been shown to enhance glycemic control by reducing glycosylated haemoglobin and improving both fasting and postprandial glucose levels compared to other antidiabetic agents [9]. Tirzepatide also helps reduce body weight and offers additional cardiovascular benefits by improving lipid profile, lowering blood pressure and decreasing visceral fat [10]. In addition, Sodium‐glucose cotransporter‐2 inhibitors (SGLT2i) provide cardiovascular benefits, such as promoting natriuresis, reducing fluid overload, improving renal haemodynamics, decreasing inflammation and enhancing myocardial energetics. These agents also reduce major adverse cardiovascular outcomes and the risk of hospitalization for heart failure [11]. SGLT‐2i are also utilized to reduce body weight and improve glycemic control, thereby lowering blood glucose levels [12]. The SUGAR‐DM‐HF and EMPA‐TROPISM trials enrolled a total of 189 patients. They demonstrated a significant reduction in left ventricular end‐diastolic volume (LVEDV) in the SGLT2i group compared to placebo [13]. In STEP‐HFpEF, semaglutide produced significantly greater improvements in KCCQ Clinical Summary Score and body weight than placebo in patients with obesity‐related HFpEF without diabetes [14]. STEP‐HFpEF DM extended these findings to patients with concurrent type 2 diabetes, again showing greater symptom and weight benefit with semaglutide than placebo [15].

Although tirzepatide and SGLT2i have shown individual benefits in managing the metabolic and haemodynamic factors of HFpEF, it is still important to compare their effectiveness and explore if they work better together. This narrative review provides a comprehensive overview of the potentially synergistic roles of tirzepatide and SGLT2 inhibitors in the management of obesity‐related HFpEF. By synthesizing high‐impact evidence spanning from 2015 to the most recent clinical advancements of 2026, this paper explores how these drug classes may converge to address the cardio‐renal‐metabolic axis. Rather than examining these therapies in isolation, we evaluate how their combined use could offer a more integrated approach to addressing cardiovascular risks and improving quality of life.

## 2 Mechanistic Pathways in Obesity‐Related HFpEF: Adiposity/EAT, Systemic Inflammation, Microvascular Dysfunction, Renal Congestion and Myocardial Energetics

Obesity has emerged as the dominant comorbidity shaping the contemporary HFpEF phenotype. Still, it is the distribution and biological quality of adipose tissue—rather than body mass index per se—that translate excess adiposity into the pathobiology of HFpEF [16]. In particular, visceral and epicardial adipose tissue are recognized as the upstream drivers of disease, because EAT lies in direct anatomical and microvascular continuity with the myocardium. When it becomes inflamed, it releases fibroinflammatory mediators that promote pericardial restraint, intramyocardial lipid infiltration, endothelial dysfunction, cardiomyocyte stiffening and left ventricular hypertrophy [17]. Notably, the high‐pressure, exercise‐intolerant HFpEF phenotype can be recapitulated by an inflamed EAT depot even in the absence of overt systemic obesity, underscoring EAT as a mechanistic hub rather than a passive fat store [18]. Inflamed EAT and visceral adipose tissue sustain a state of chronic low‐grade systemic inflammation—a metaflammation in which circulating tumour necrosis factor‐α, interleukin‐6 and high‐sensitivity C‐reactive protein not only are elevated but also correlate with greater comorbidity burden, worse invasive haemodynamics and right‐ventricular dysfunction in patients with HFpEF [19]. Critically, this inflammatory state is not driven solely by heightened pro‐inflammatory initiation; a parallel deficiency in inflammation‐resolution programmes equally sustains it, as obese adipose tissue is characterized by inadequate biosynthesis of specialized pro‐resolving mediators (resolvins, protectins and maresins) and reduced expression of their receptors, perpetuating neutrophil and M1‐macrophage dominance, defective efferocytosis and persistent tissue maladaptation [20]. This dual imbalance—excess initiation coupled with failed resolution—provides a coherent biological substrate for the systemic inflammatory drive that underpins obesity‐related HFpEF and offers a unifying framework for therapies that engage both arms of the inflammation axis [21].

This systemic inflammatory milieu propagates downstream to the heart via coronary microvascular dysfunction, which has emerged as a central intermediate phenotype linking comorbidity‐driven inflammation to diastolic dysfunction [22]. Endothelial activation reduced nitric oxide bioavailability, impaired coronary flow reserve and capillary rarefaction curtail cardiomyocyte perfusion and depressed cyclic guanosine monophosphate–protein kinase G signalling, thereby stiffening the cardiomyocyte, impairing both active and passive lusitropy and initiating interstitial fibrosis, the structural hallmarks of obesity‐related HFpEF [23]. In parallel, the same visceral adiposity that drives EAT expansion elevates intra‐abdominal and right‐sided filling pressures, thereby reducing systemic venous compliance and increasing effective stressed blood volume that converges on the renal circulation [24]. The resulting cardiorenal syndrome is amplified by retrograde transmission of elevated central venous pressure into the renal veins and by mechanical compression of the renal parenchyma from raised intra‐abdominal pressure, establishing venous congestion as a self‐reinforcing amplifier of HFpEF severity and a therapeutic target in its own right [25]. These intermediate haemodynamic and microvascular insults converge, at the organ level, on the myocardium as a state of bioenergetic deprivation: integrated multi‐omic profiling of left‐ventricular biopsies from patients with obese‐related HFpEF demonstrates impaired glycolysis, accumulation of glucose‐6‐phosphate and succinate together with a shifted succinate‐to‐fumarate ratio and a reduced adenosine triphosphate/adenosine diphosphate ratio, changes that correlate tightly with New York Heart Association class, E/e', left‐ventricular filling pressures and attenuated stroke‐volume augmentation on exercise [26]. This metabolic signature—corroborated by complementary evidence of altered substrate utilization, oxidative phosphorylation and high‐energy‐phosphate transport in human and experimental HFpEF—co‐exists with up‐regulated extracellular matrix organization as the two cardinal axes of the cardiometabolic HFpEF phenotype [27]. Within this integrated framework, glucagon‐like peptide‐1 receptor agonists act at the convergence of all five pillars, plausibly modulating EAT and visceral adipose inflammation, attenuating metaflammation, restoring coronary endothelial reactivity and relieving lipotoxic and fibrotic stress on the energetically compromised cardiomyocyte, thereby providing a biologically grounded rationale for the clinical benefits observed in obesity‐related HFpEF [28].

## 3 Tirzepatide for Heart Failure With Preserved Ejection Fraction and Obesity

### 3.1 Tirzepatide: Mechanisms of Action in Obesity–Related HFpEF

GLP‐1 (glucagon‐like peptide‐1) has been a primary focus for the treatment of type 2 diabetes and obesity since its discovery in 1987. GLP‐1 is released from the gut after nutrient ingestion and increases insulin secretion in response to glucose, helping regulate blood sugar levels [29, 30]. The glucose‐dependent insulinotropic peptide (GIP) was one of the two main first incretin hormones discovered [31, 32] along with GLP‐1, secreted by K‐cells in the duodenum, stimulating glucose‐mediated insulin secretion. The combination of GIP with synthetic GLP‐1 receptor agonists produced additive effects on both glucose regulation and body weight [32]. These trials established that intentional weight loss with a GLP‐1 receptor agonist meaningfully improves symptoms and function in obesity‐related HFpEF. The SUMMIT trial subsequently extended this paradigm to dual GIP/GLP‐1 receptor agonism with tirzepatide, demonstrating reductions in worsening heart failure events alongside similar improvements in weight and quality of life [10].

**FIGURE 1:**
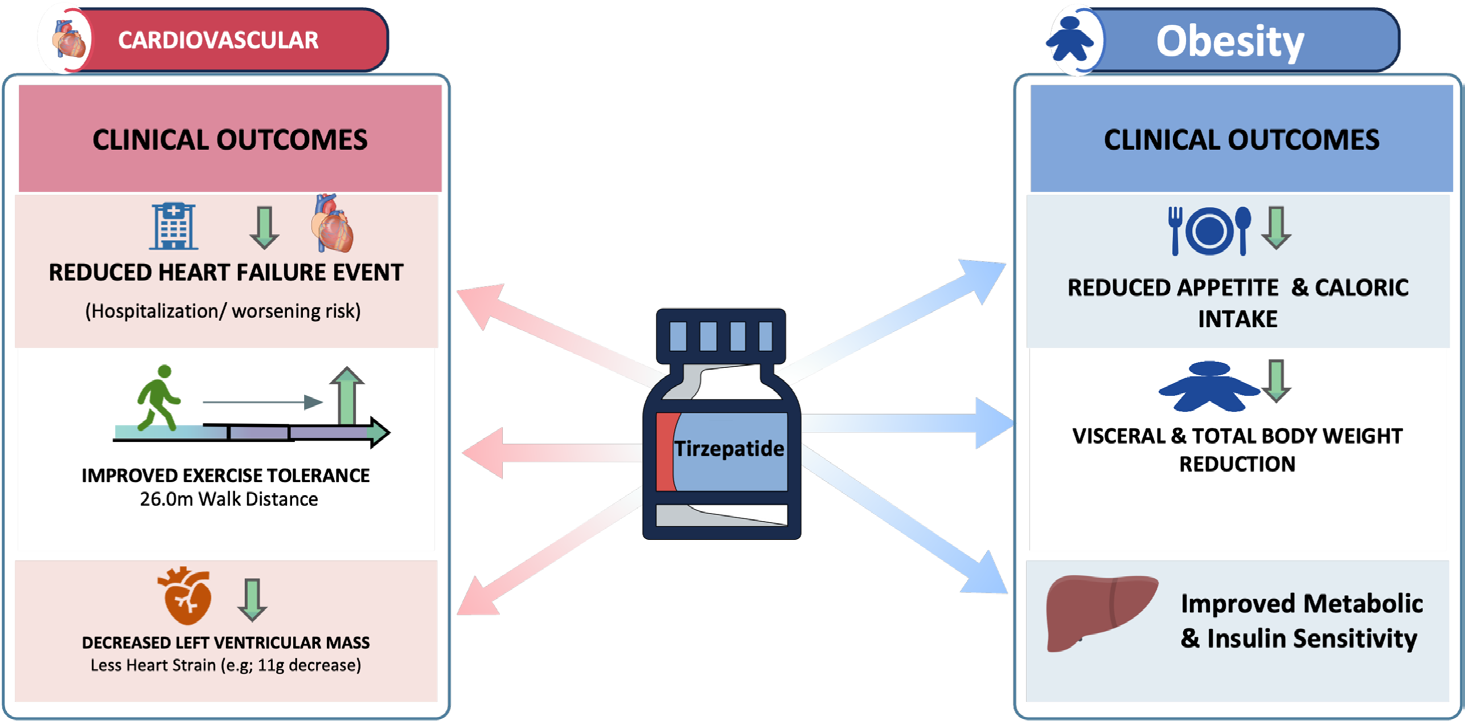
Dual cardiovascular and metabolic outcomes of tirzepatide therapy. Tirzepatide administration drives complementary clinical benefits across two primary axes. The cardiovascular panel outlines reductions in heart failure events, enhanced exercise capacity and structural offloading via decreased left ventricular mass. The metabolic panel details systemic obesity management, driven by caloric reduction, profound visceral and total body weight loss and enhanced insulin sensitivity.

Tirzepatide functions as a dual GIP/GLP‐1 receptor agonist, exhibiting high potency at the GIP receptor and substantial activity at the GLP‐1 receptor [20]. Within pancreatic islets, tirzepatide enhances the incretin effect. Activation of the GLP‐1 receptor significantly elevates β‐cell cAMP and promotes glucose‐dependent insulin secretion, while indirectly suppressing α‐cell glucagon secretion through intra‐islet Somatostatin during hyperglycemia [33, 34]. In addition, GLP‐1‐induced release of atrial natriuretic peptide (ANP) and suppression of renal angiotensin II further enhance salt excretion, thereby reducing intravascular volume and blood pressure [34]. Concurrent activation of the GIP receptor enhances glucose‐stimulated insulin release via the AKT/PKB signalling pathway. It also promotes β‐cell proliferation and prevents apoptosis, which is critical for maintaining glycemic control [34, 35]. By dual activating GLP‐1 and GIP signalling pathways, which synergistically enhance insulin secretion and inhibit glucagon secretion, improving glucose homeostasis is highly effective. Furthermore, dual receptor agonists exert more pronounced synergistic effects in suppressing appetite and promoting satiety [35]. The SUMMIT trial demonstrated that tirzepatide therapy in patients with obesity‐related heart failure with preserved ejection fraction (HFpEF) resulted in reduced left ventricular (LV) mass and paracardiac adipose tissue (PAT) [10]. GIP receptors show high expression in adipose tissue. GIP signalling upregulates lipoprotein lipase and enhances triglyceride uptake, thereby promoting efficient storage of dietary lipids in metabolically healthy adipocytes [34, 36]. In contrast, GLP‐1 has very little direct receptor expression in adipocytes. Most of its effects happen indirectly through autonomic and metabolic pathways, such as increased sympathetic activity that helps break down stored triglycerides [34, 37, 38]. Together, these complementary actions enhance adipocyte function. Tirzepatide increases insulin sensitivity, adiponectin secretion and the lipid‐buffering capacity of subcutaneous fat and reduces ectopic lipid deposition in muscle, liver and myocardium and improves systemic insulin resistance [37, 38]. In the central nervous system, tirzepatide suppresses appetite, promotes satiety, reduces vomiting and facilitates weight loss [17]. In the liver, this agent increases insulin sensitivity, inhibits hepatic gluconeogenesis and influences ectopic lipid accumulation in non‐adipose tissues [30]. The integrated clinical benefits of tirzepatide across these cardiovascular and metabolic domains are summarized in Figure 1.

### 3.2 Metabolic and Weight‐Reduction Efficacy of Tirzepatide

Early research involving individuals with obesity and prediabetes examined whether lifestyle interventions [39, 40], pharmacotherapies, or bariatric surgery could prevent or delay the onset of type 2 diabetes [40]. Later studies focused on returning to normal blood sugar levels, since prediabetes can lead to several health problems, such as microvascular and macrovascular complications [40]. Tirzepatide therapy has led to significant weight loss, better blood sugar control and improvements in other metabolic measures for people with obesity, whether they have type 2 diabetes [36, 41].

During the initial 72 weeks of the SURMOUNT‐1 trial (the primary period of this trial), participants with obesity who received a 15‐mg dose of tirzepatide experienced an average weight loss exceeding 20% of total body weight, along with a 0.51% reduction in glycated haemoglobin levels [41]. However, people who have both obesity and type 2 diabetes often lose less weight when treated with anti‐obesity medications. According to the SURMOUNT‐2 trial, individuals with obesity and type 2 diabetes (T2D) who received a 15‐mg dose of tirzepatide achieved an average weight loss of 14.7% of total body weight and a 2.1% reduction in glycated haemoglobin levels [36]. Furthermore, tirzepatide has shown profound efficacy in preventing T2D. In a double‐blind, randomized controlled trial, only 1.3% of participants in the pooled tirzepatide group developed T2D by week 176, compared to 13.3% in the placebo group [37].

Tirzepatide therapy also yields a meaningful improvement in metabolic dysfunction–associated steatosis liver disease (MASLD), previously classified as nonalcoholic fatty liver disease (NAFLD), which is highly prevalent in the obese population [32, 42]. For patients with type 2 diabetes, tirzepatide treatment reduced liver fat and improved biomarkers linked to Metabolically Dysfunctional‐Associated Steatohepatitis (MASH) and fibrosis [42]. In the SYNERGY‐NASH trial, 55% of participants in the 5‐mg tirzepatide group demonstrated at least one stage of improvement in fibrosis without a worsening of MASH, compared to only 30% in the placebo group [42]. Additionally, the percentage of participants who met the criteria for resolution of MASH without worsening of fibrosis was significantly higher in all three tirzepatide groups compared to the placebo group [42]. Beyond hepatic metrics, tirzepatide also reduced waist size and visceral fat more than the placebo, with the 5, 10 and 15 mg doses resulting in 15.0%, 19.5% and 20.9% reductions in total fat mass, respectively [41, 43].

### 3.3 Tirzepatide for HFpEF Treatment

Tirzepatide may enhance cardiovascular health through several mechanisms. It helps control blood sugar by lowering HbA1c levels, which reduces the risk of vascular damage linked to high blood sugar. High blood glucose and fluctuations can lead to problems with blood vessel function and atherosclerosis [40]. Tirzepatide significantly reduced infiltration of pro‐inflammatory M1 adipose tissue macrophages and lowered levels of inflammatory cytokines, thereby improving insulin sensitivity [44, 45]. The study demonstrates that tirzepatide reduced circulatory volume, measured by blood volume (BV) and plasma volume (PV), after 12 weeks of treatment. It also reduced systolic blood pressure (BP), with this change seen after 4 weeks [46]. Tirzepatide treatment led to significant improvements in atherogenic lipids. Total cholesterol decreased by 3.8%, 4.6% and 5.9% at doses of 5, 10 and 15 mg. Triglycerides and LDL‐C were also reduced, while HDL‐C increased [47]. Tirzepatide can boost NO production and increase endothelial NO synthase activity in endothelial cells [48].

The SUMMIT trial found that tirzepatide lowered the risk of worsening heart failure in patients with obesity‐related HFpEF compared to placebo. It also reduced heart failure symptoms and improved exercise tolerance [10]. A retrospective analysis further indicated that patients in the tirzepatide group had a much lower rate of major adverse cardiovascular events (MACE) and heart failure exacerbations (HFE), with rates of 2.64 compared to 5.05 per 100 person‐years in the control group [49]. Crucially, as shown in the SUMMIT CMR substudy, the tirzepatide group exhibited a significant reduction in epicardial adipose tissue (EAT) and paracardiac adipose tissue (PAT) compared to the placebo group, alongside an approximate 11 g decrease in left ventricular mass [6]. The SURPASS‐CVOT study similarly highlighted cardiovascular safety and potential benefit, noting that tirzepatide reduced the risk of MACE‐3 by 8% (HR = 0.92 [95.3% CI 0.83–1.01]) and all‐cause mortality by 16% (HR = 0.84 [95.0% CI 0.75–0.94]) compared to Dulaglutide [8]. Overall, tirzepatide significantly reduced heart failure events requiring hospitalization or urgent treatment. The increase in 6‐min walk distance was 26.0 m in the tirzepatide group compared with 10.1 m in the placebo group. Additionally, the systemic inflammatory marker high‐sensitivity C‐reactive protein (CRP) decreased by a remarkable 38.8% in the tirzepatide group. In contrast, the placebo group experienced only a 5.9% reduction, corresponding with a 4.6 mmHg greater reduction in systolic blood pressure [10].

## 4 SGLT2 Inhibitors: Foundational Therapy for HFpEF

(SGLT2i) have become a transformative class in cardiovascular medicine. Originally established for glycemic control in type 2 diabetes, SGLT2i have demonstrated powerful cardiovascular and renal protective effects, independent of baseline glycemic control [11]. These findings indicate that SGLT2i offer multiple pleiotropic benefits, including induced natriuresis, decreased fluid overload, improved renal haemodynamics, attenuated systemic inflammation and enhanced myocardial energetics [11]. As a result, international guidelines now strongly recommend SGLT2i as foundational therapy for heart failure, regardless of ejection fraction or diabetes status [11, 40]. While initially utilized as a glucose‐lowering medication, their role in reducing major adverse cardiovascular outcomes and hospitalizations for heart failure has been definitively established. The clinical impact of these agents has subsequently been shown to be largely uncoupled from their glucose‐lowering magnitude, prompting several hypotheses regarding their exact mechanisms of action [13]. Over the past few decades, heart failure morbidity and mortality have decreased due to the implementation of therapies targeting neurohormonal pathophysiology, including beta‐blockers, angiotensin‐converting enzyme inhibitors (ACEI), angiotensin receptor blockers (ARB), angiotensin receptor‐neprilysin inhibitors (ARNI) and mineralocorticoid receptor antagonists (MRA) [50]. Nevertheless, this benefit has historically been restricted to heart failure with reduced ejection fraction (HFrEF) rather than HFpEF [50]. Fortunately, hospitalizations for both HFrEF and HFpEF have decreased since the introduction of SGLT2i [50].

**FIGURE 2:**
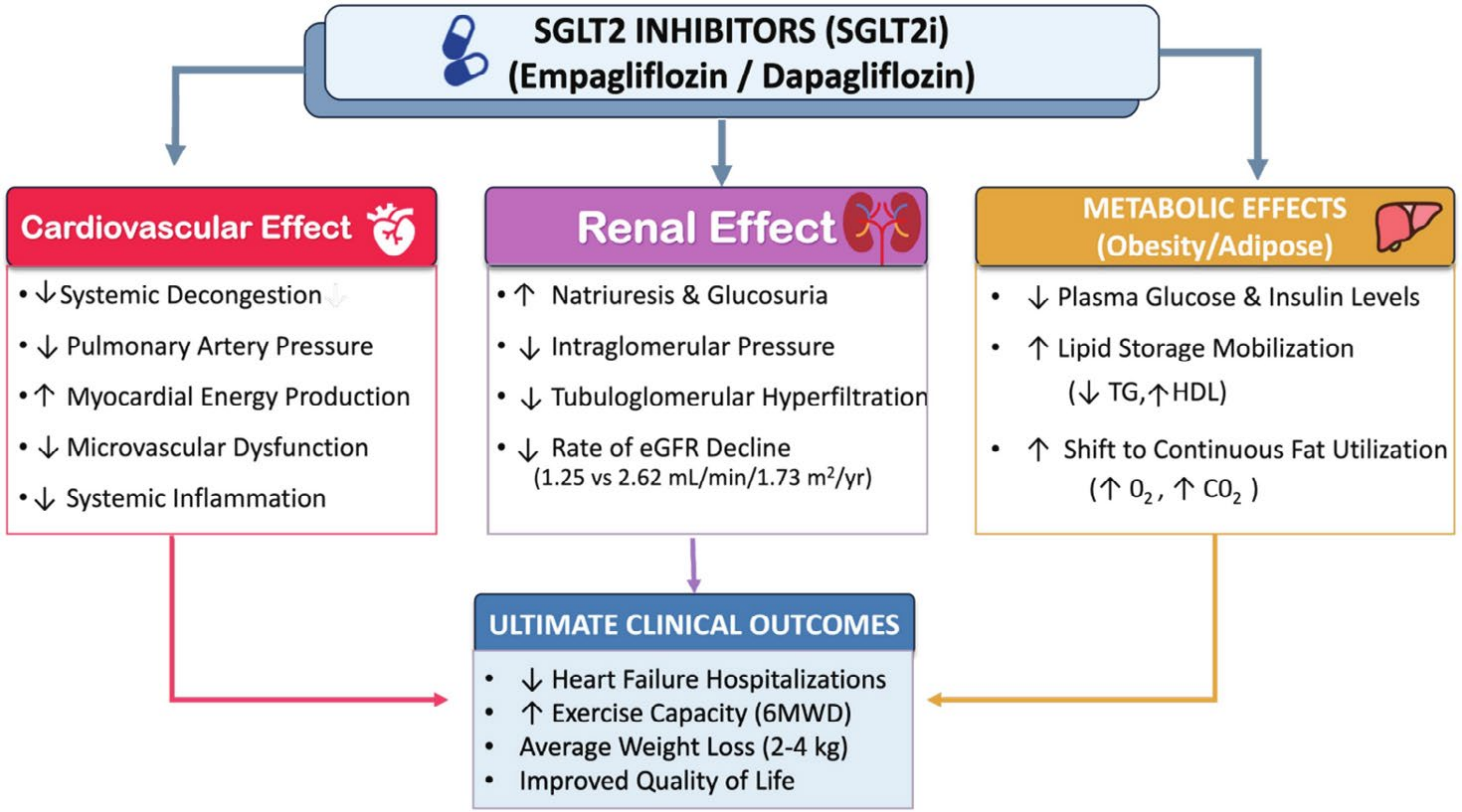
Pleiotropic mechanisms and clinical outcomes of SGLT2 inhibitors (Empagliflozin and Dapagliflozin). The schematic illustrates the primary cardiovascular, renal and metabolic pathways modulated by SGLT2 inhibition. These combined systemic effects directly translate into favourable ultimate clinical outcomes, including reduced heart failure hospitalizations, increased exercise capacity, targeted weight loss and an overall improved quality of life.

Several plausible pathways could explain the therapeutic benefits of SGLT2 inhibitors found in recent clinical trials (Figure 2). First, SGLT2i have been shown to rapidly lower pulmonary artery pressure, thereby facilitating systemic decongestion and leading to clinical improvements in symptoms and exercise capacity [51, 52]. Second, SGLT2i may increase myocardial energy production, reduce systemic microvascular dysfunction (which is prevalent in both the myocardium and skeletal muscle in HFpEF), restore systemic endothelial function, decrease systemic inflammation and oxidative stress, improve insulin sensitivity and activate fatty acid oxidation in skeletal muscle [53, 54, 55, 56]. In a pivotal trial evaluating Dapagliflozin, the agent was compared with a placebo in individuals with either preserved or mildly reduced ejection fraction (LVEF > 40%) [57]. Dapagliflozin treatment significantly enhanced submaximal exercise capacity, determined by a 6‐min walk distance and patient‐reported health status, with patients having a higher body mass exhibiting greater clinical improvements [58]. Furthermore, a study of 84 patients found that Empagliflozin improved left ventricular function, cardiac remodelling and quality of life compared with placebo. Conversely, the EMPIRE‐Study, which included 190 diabetic and non‐diabetic patients, found no significant difference between Empagliflozin and placebo for select endpoints (including NT‐proBNP levels, activity level and symptomatic clinical improvement) at 3 months post‐initiation [13]. Additionally, the EMPEROR‐Preserved trial highlighted the renal‐protective effects of Empagliflozin. Patients on Empagliflozin experienced a significantly slower decline in their estimated glomerular filtration rate (eGFR) than those on a placebo; specifically, the Empagliflozin cohort experienced an annual eGFR decline of 1.25 mL/min/1.73 m^2^, whereas the placebo cohort experienced a decline of 2.62 mL/min/1.73 m^2^ [59].

Obesity is defined as an abnormal or excessive accumulation of fat in adipocytes and poses a severe cardiovascular health risk [60]. The primary form of fat storage in adipose tissue is triglycerides. Excessive release of free fatty acids (FFAs) from the lipolysis of visceral adipose tissue into the circulation or the portal vein impairs the function of vital organs such as the heart, liver and kidneys. Therefore, decreasing triglyceride accumulation or promoting fat utilization in adipose tissue is a primary approach to mitigating obesity‐related risks [60]. SGLT2i reduces plasma glucose and insulin levels while promoting lipid storage mobilization [61]. Treatment with SGLT2i has been associated with reduced serum triglycerides, increased HDL cholesterol and a slight, clinically manageable increase in LDL cholesterol [62].

Mechanistically, Empagliflozin increases energy expenditure, evidenced by increased carbon dioxide exhalation and oxygen consumption, thereby shifting substrate metabolism towards increased fat and glucose utilization [60]. Clinically, SGLT2i treatment can result in an average body weight reduction of 2 to 4 kg [63]. Chronic administration of Empagliflozin directs the body toward continuous fat utilization, accompanied by decreased tissue glucose disposal. These findings suggest that Empagliflozin suppresses weight gain by shifting systemic energy metabolism [60]. Furthermore, administration of Licogliflozin at 150 mg once daily and 50 mg twice daily resulted in weight reductions of 4.26% and 5.2%, respectively, in normoglycemic subjects with obesity compared to placebo. Significant changes in HbA1c after 24 weeks were observed only with the highest dose regimens of Licogliflozin in patients with T2DM [64].

## 5 Clinical Efficacy and Synergistic Potential of Tirzepatide and SGLT2 Inhibitors

Tirzepatide and SGLT2i both demonstrate benefits in obesity‐related heart failure with preserved ejection fraction (HFpEF). Tirzepatide lowered the rates of death and hospitalization in patients with obesity‐related HFpEF. The main outcome, which included death from heart‐related causes or worsening heart failure, occurred in 36 patients (9.9%) taking tirzepatide and 56 patients (15.3%) in the placebo group [10]. In the EMPEROR‐Preserved trial, the main combined outcome of death from cardiovascular causes or hospitalization for heart failure occurred in 415 patients (13.8%) in the Empagliflozin group and 511 patients (17.1%) in the placebo group [65]. The group treated with tirzepatide had a significant reduction in epicardial adipose tissue (EAT) and paracardiac adipose tissue (PAT) compared to the placebo group [6]. At 52 weeks, patients treated with Empagliflozin demonstrated greater improvement in Kansas City Cardiomyopathy Questionnaire (KCCQ) clinical summary scores compared to those receiving placebo (4.51 vs. 3.18) [65]. In contrast, patients receiving tirzepatide exhibited a 19.5‐point improvement, whereas the placebo group showed a 12.7‐point increase [10]. Because SUMMIT and EMPEROR‐Preserved enrolled different populations, used different eligibility criteria, had different follow‐up durations and tested different interventions against their own placebo arms, direct cross‐trial comparison is not statistically valid. Table 1 therefore summarizes evidence from each pivotal trial separately rather than comparing the two drug classes head‐to‐head.

**TABLE 1:** Summary of Evidence from Pivotal Trials of Tirzepatide (SUMMIT) and SGLT2 Inhibitors (EMPEROR‐Preserved) in Obesity‐Related HFpEF.

Feature/Category	Population	Follow‐up	Tirzepatide	SGLT2 inhibitors (Empagliflozin)	References
HFpEF Primary Outcome (Death/Hospitalization)	SUMMIT: *n* = 731, BMI ≥ 30, LVEF ≥ 50%, NYHA II–IV	SUMMIT: ~2 years (event‐driven); EMPEROR‐Preserved: median 26.2 months	SUMMIT Trial (9.9%)	EMPEROR‐Preserved Trial (13.8%)	[10, 65]
KCCQ Clinical Score Improvement	SUMMIT: *n* = 731, BMI ≥ 30, LVEF ≥ 50%, NYHA II–IV	SUMMIT: 52 weeks; EMPEROR‐Preserved: 52 weeks	SUMMIT Trial (19.5%)	EMPEROR‐Preserved Trial (4.51%)	[10, 65]
MASLD	SYNERGY‐NASH: T2D + MASH/fibrosis cohort; SGLT2i: T2D + MASLD cohort	SYNERGY‐NASH: 52 weeks	Significantly reduced fatty liver and 55% improve by at least one stage of liver fibrosis without worsening MASLD	Reduced liver fat and fibrosis	[42, 66]
Adipose Tissue (EAT/PAT)	SUMMIT CMR substudy: *n* = 106, obesity‐related HFpEF; SGLT2i EAT studies: smaller mechanistic cohorts	SUMMIT CMR substudy: 52 weeks	Significant reduction in EAT and PAT	Significant reduction in epicardial adipose tissue (EAT)	[6, 67]
Weight Reduction	SURMOUNT trials: obesity ± T2D (distinct cohort from SUMMIT/EMPEROR‐Preserved)	SURMOUNT‐1: 72 weeks	Significant reduction (more than 20%)	Middle reduction in weight and waist circumference	[41, 68]
Volume effect	SUMMIT secondary analysis: *n* = 731, obesity‐related HFpEF	SUMMIT: 12 weeks. (BV/PV); 52 weeks. (BP)	Decrease in circulatory volume	Decrease in extracellular volume	[46, 67]
Glycated Haemoglobin	SURMOUNT trials: obesity ± T2D	SURMOUNT‐1/2: 72 weeks	Decreased levels	Decreased levels	[36, 41, 63]
Serum lipid profile	Tirzepatide meta‐analysis (mixed T2D/obesity); SGLT2i lipid studies (mixed cohorts)	Variable by study, 24–52 weeks	Decreased total cholesterol, LDL‐C, triglycerol and increase HDL‐C	Decrease serum triglyceride, increase HDL, Cholesterol and slightly increase LDL.	[47, 66]

Abbreviations: EAT, epicardial adipose tissue; HDL, high density lipoprotein; HFpEF, heart failure with preserved ejection fraction; KCCQ, Kansas City Cardiomyopathy Questionnaire; LDL, low density lipoprotein; MASLD, metabolic dysfunction‐associated steatotic liver disease; PAT, Paracardial adipose tissue; TG, triglyceride.

Tirzepatide demonstrates a beneficial effect in the management of obesity. Administration of tirzepatide resulted in a reduction in body weight of more than 20% and decreased glycated haemoglobin (HbA1c or A1C) levels [41]. While incretin therapies like tirzepatide directly target adiposity pathways, SGLT2 inhibitors provide vital haemodynamic offloading that functions independently of baseline body weight. Subgroup analyses from major trials confirm that the clinical efficacy of dapagliflozin in reducing heart failure events remains entirely consistent across the full spectrum of patient body mass indexes, including those with severe obesity [69]. GLP‐1 receptor agonists, when administered as monotherapy, result in greater weight loss [68]. Empagliflozin, when compared to placebo, resulted in modest reductions in weight, waist circumference and both systolic and diastolic blood pressure, without an increase in heart rate [70]. Tirzepatide therapy resulted in significant improvement in MASLD. Specifically, 30% of participants in the placebo group and 55% in the 5 mg tirzepatide group demonstrated at least one stage of improvement in fibrosis without worsening of MASH. Additionally, tirzepatide reduced liver fat and improved biomarkers associated with MASH and fibrosis [42]. Empagliflozin significantly reduced liver fat compared to the control group [66]. In the Empagliflozin group, aspartate aminotransferase (AST) levels decreased and liver fibrosis was significantly reduced [67]. Tirzepatide reduced total cholesterol by 3.8%, 4.6% and 5.9% at doses of 5, 10 and 15 mg, respectively. Triglyceride and low‐density lipoprotein cholesterol (LDL‐C) levels also decreased, while high‐density lipoprotein cholesterol (HDL‐C) increased [47]. In contrast, SGLT2i did not significantly alter LDL‐C or triglyceride (TG) levels in any group. Some studies comparing them to other antidiabetic agents, such as pioglitazone, have shown a significant increase in HDL‐C [67]. The Empagliflozin group exhibited a significant reduction in epicardial adipose tissue (EAT) and a 1.25% greater decrease in extracellular volume compared to the placebo group [71]. Additionally, tirzepatide reduced circulatory volume, as measured by blood volume (BV) and plasma volume (PV) [46].

**FIGURE 3:**
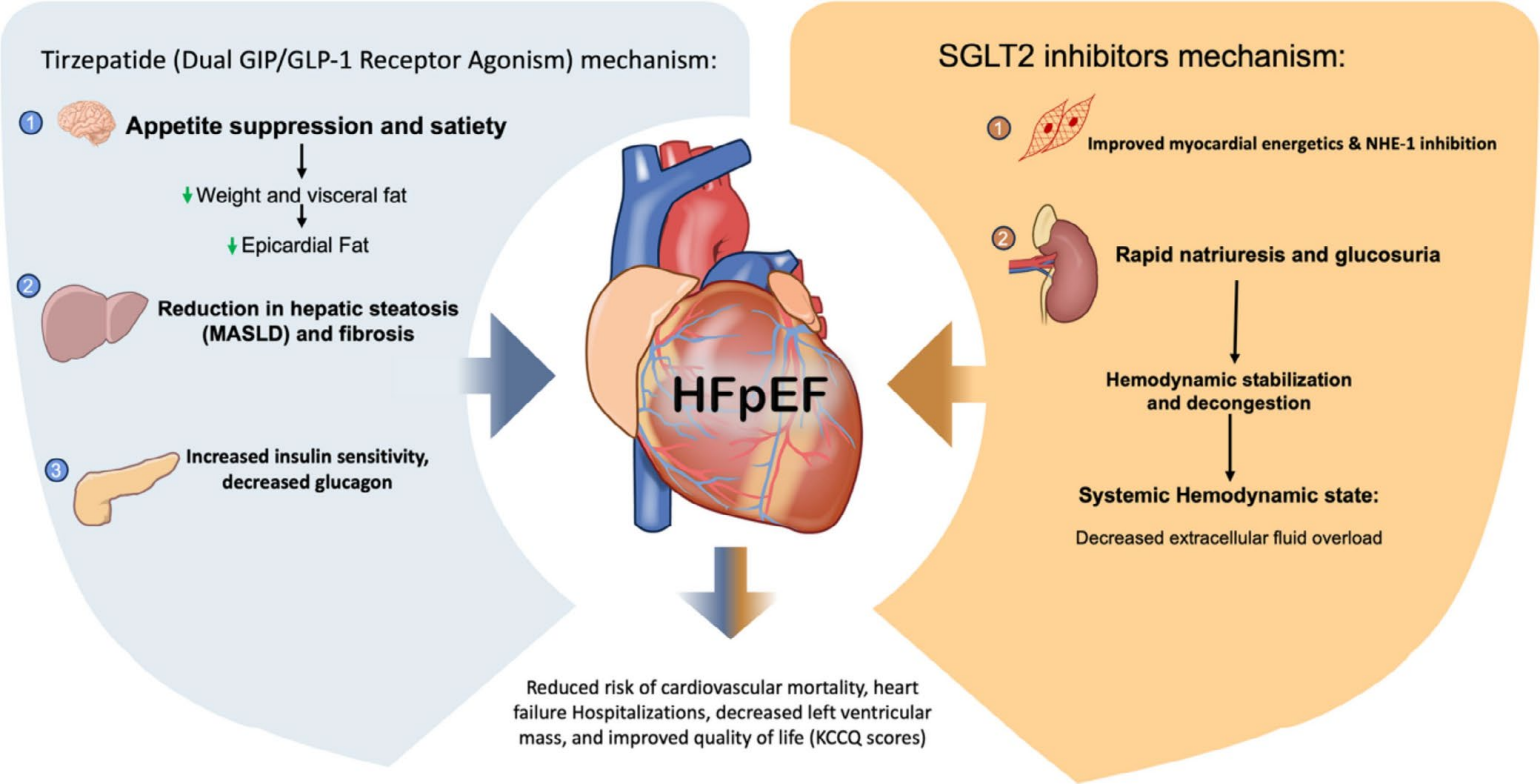
Potential synergistic mechanisms of tirzepatide and SGLT2i in the management of obesity‐related HFpEF. The diagram illustrates the distinct but complementary physiological axes of both therapies. The metabolic pathway of dual GIP/GLP‐1 agonism is characterized by central appetite suppression, multi‐organ fat reduction (including epicardial adipose tissue) and enhanced insulin sensitivity, while the haemodynamic and energetic pathway of SGLT2 inhibition features direct myocardial energetic improvements and systemic decongestion driven by rapid natriuresis. Together, these parallel pathways converge on the failing heart to reduce worsening heart failure events, decrease hospitalizations and improve patient quality of life.

The combination of SGLT2i and tirzepatide has the potential to deliver synergistic benefits by targeting these complementary physiological axes while converging on a critical shared target: the reduction of epicardial adipose tissue (EAT) (see Figure 3). Tirzepatide drives profound metabolic recovery through substantial weight loss, while SGLT2 inhibitors act as a cornerstone for haemodynamic stability through rapid fluid offloading. Together, these therapies offer a more comprehensive approach to managing obesity‐related HFpEF. However, because dedicated clinical trials evaluating this specific combination are lacking, further research is needed to establish its long‐term safety and practical efficacy. The current evidence hierarchy should be stated explicitly: randomized evidence supports each drug class individually, whereas data on their combined use in HFpEF remain largely observational or extrapolated. A retrospective target‐trial‐emulation analysis of patients with overweight/obesity, type 2 diabetes and obesity‐related HFpEF already receiving SGLT2 inhibitors found additional benefit associated with GLP‐1 receptor agonist use, but its retrospective design precludes causal conclusions [72].

Combining tirzepatide with SGLT2 inhibitors represents a physiologically rational strategy. This approach effectively targets the interconnected issues of the heart, kidney and metabolism through distinct yet complementary mechanisms. While SGLT2 inhibitors help quickly stabilize blood flow by promoting the excretion of salt and reducing fluid overload, tirzepatide addresses the underlying metabolic issues linked to the progression of heart failure with preserved ejection fraction (HFpEF), especially excess fat around organs. Notably, tirzepatide has been shown to reduce paracardiac adipose tissue in obesity‐related HFpEF [6]. In contrast, empagliflozin independently reduces epicardial adipose tissue volume in patients with heart failure [71]. There's a growing belief that combining these treatments could effectively reduce key issues related to heart failure with preserved ejection fraction (HFpEF), especially in patients dealing with obesity. However, this idea still needs to be tested in future studies. So far, there hasn't been a dedicated randomized controlled trial specifically evaluating this combination in HFpEF patients with obesity. Still, the way these drugs work together and their different side effects make the combination seem promising. The 2023 update to the European Society of Cardiology's guidelines on heart failure emphasizes a tailored multi‐drug approach for high‐risk HFpEF patients. This supports the idea that combining these two drug classes is a reasonable strategy, although more research is needed to confirm its effectiveness.

### 5.1 Safety Profile and Adverse Effects of Tirzepatide and SGLT2 Inhibitors

The safety profile of tirzepatide, as established across the SURMOUNT and SURPASS trial programs, is generally favourable; however, clinicians must remain vigilant regarding several adverse effects [41]. Gastrointestinal events represent the most frequently reported adverse effects, including nausea, vomiting, diarrhoea and constipation, predominantly occurring during dose escalation and typically transient in nature. These effects are largely attributed to delayed gastric emptying mediated by GLP‐1 receptor activation [41]. More serious, albeit rare, adverse events include acute pancreatitis and gallbladder or biliary disease; a systematic review and meta‐analysis demonstrated that tirzepatide was associated with an increased risk of composite gallbladder or biliary disease (RR 1.97, 95% CI 1.14–3.42) compared with placebo or basal insulin, though no statistically significant increase in pancreatitis risk was confirmed (RR 1.46, 95% CI 0.59–3.61) [73, 74]. Of particular clinical concern in the obesity‐related HFpEF population is the risk of lean muscle mass loss accompanying significant weight reduction [28], which may impair functional capacity if not managed with adequate protein intake and supervised resistance exercise [75, 76, 77]. Furthermore, tirzepatide carries a boxed warning regarding the risk of thyroid C‐cell tumours observed in rodent models and is contraindicated in patients with a personal or family history of medullary thyroid carcinoma or MEN2 [41]. In keeping with the general principles of individualized care emphasized in the American Diabetes Association (ADA) Standards of Care 2025, risk–benefit assessment should be undertaken prior to initiating tirzepatide in elderly or frail obesity‐related HFpEF patients [78].

**FIGURE 4:**
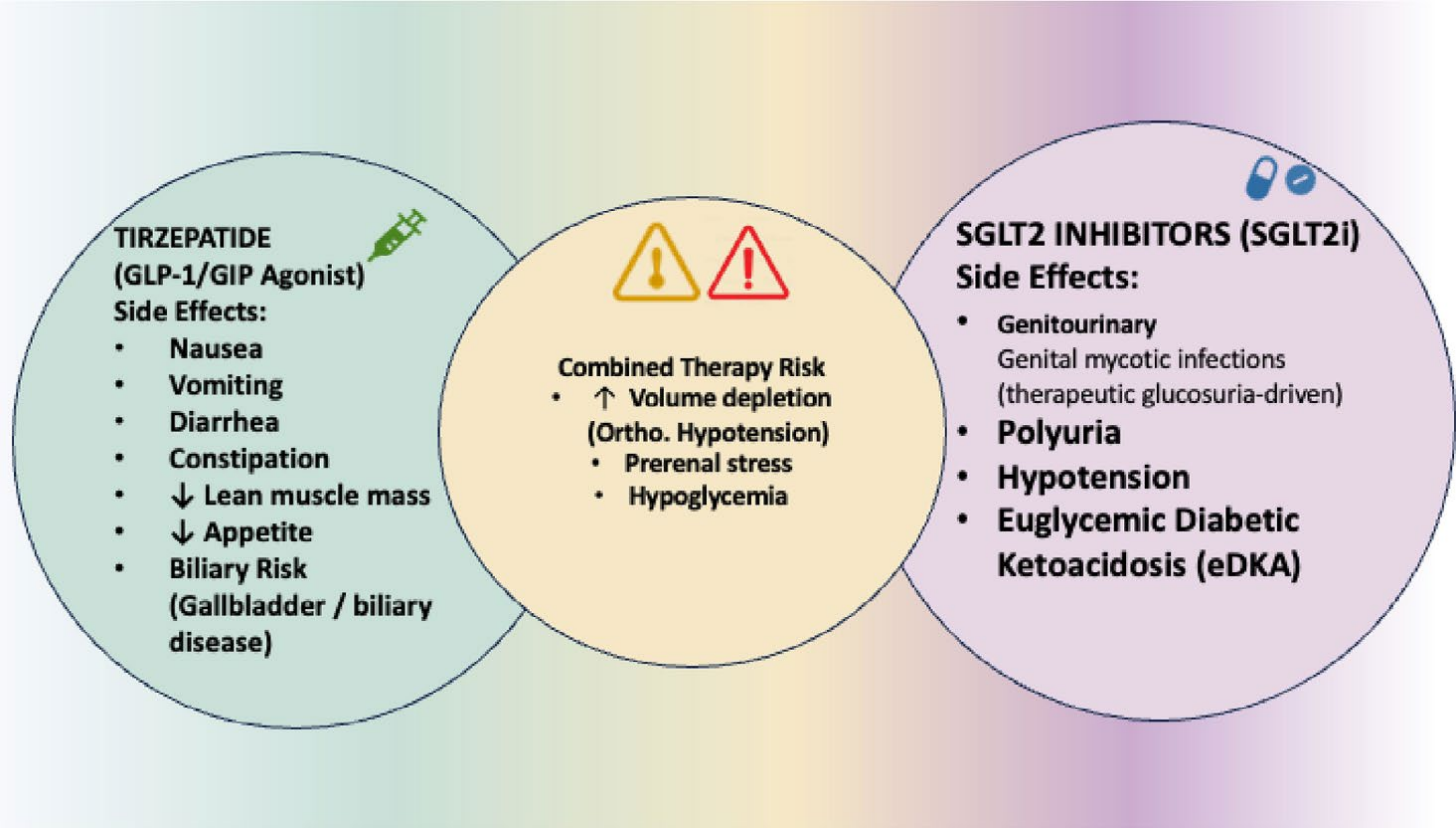
Individual and potential compounded adverse effect profiles of tirzepatide and SGLT2 inhibitors. The outer sections delineate the established adverse events unique to each pharmacological class, including gastrointestinal disturbances and lean muscle reduction for tirzepatide, alongside genitourinary infections and euglycemic diabetic ketoacidosis (eDKA) for SGLT2 inhibitors. The intersecting centre highlights the theoretical, yet clinically significant, risks associated with their concurrent use. Because the safety profile of this specific combination is not yet definitively established in dedicated clinical trials, the overlap illustrates anticipated physiological interactions, namely exacerbated volume depletion, orthostatic hypotension, prerenal stress and hypoglycemia that necessitate vigilant clinical monitoring.

SGLT2 inhibitors are generally well tolerated across broad patient populations, including those with HFpEF, diabetes and chronic kidney disease; however, their mechanism of action predisposes patients to a distinct adverse effect profile that warrants careful clinical consideration. Glucosuria‐induced changes in the urogenital microenvironment increase susceptibility to genitourinary infections; however, of these, only genital mycotic infections have been consistently associated with SGLT2 inhibitor use across clinical trials and observational studies, while urinary tract infections are not significantly increased by SGLT2 inhibitor use independent of the underlying disease [78]. The risk of genital mycotic infections is further amplified by diabetes‐related glucosuria, making it the principal drug‐attributable genitourinary adverse effect of this class [79, 80]. Of greater clinical concern is the risk of euglycemic diabetic ketoacidosis (DKA), which can occur in the absence of marked hyperglycemia and necessitates temporary drug discontinuation perioperatively or during prolonged fasting [81]. In practice, SGLT2 inhibitors should be withheld for at least 3 days prior to scheduled surgery and during acute illness with reduced oral intake and tirzepatide dose escalation should proceed no faster than every 4 weeks with dose‐hold or reduction if gastrointestinal intolerance limits oral intake, to reduce the risk of compounded volume depletion. Volume depletion and symptomatic hypotension may also occur, particularly when SGLT2i are co‐administered with loop diuretics, a combination frequently encountered in obesity‐related HFpEF management. Rare but serious adverse events include Fournier's gangrene (necrotizing fasciitis of the genitoperineal region) and, specifically with canagliflozin, an increased risk of lower limb amputation not consistently observed with empagliflozin or dapagliflozin [79]. (See Figure 4 for a comparative summary of these side effects.)

While the individual safety profiles of tirzepatide and SGLT2 inhibitors are well‐established, their concurrent use requires careful consideration of overlapping physiological effects. Specifically, the gastrointestinal events and potential for reduced oral fluid intake associated with tirzepatide may compound the volume depletion and osmotic diuresis induced by SGLT2 inhibitors. Consequently, co‐administration necessitates vigilant monitoring of fluid status and blood pressure to prevent additive symptomatic hypotension and excessive prerenal stress. Therefore, dedicated prospective clinical trials are warranted to definitively characterize the safety profile and establish optimal monitoring protocols for this combination therapy in the obesity‐related HFpEF population.

From a practical standpoint, candidates for combined therapy are patients with obesity‐related HFpEF and persistent symptoms or fluid overload despite guideline‐directed management, consistent with the populations enrolled in the pivotal SGLT2 inhibitor and tirzepatide HFpEF trials [10, 65, 69]. Before initiation, baseline renal function, volume status and current diuretic dose should be reviewed, in line with general heart failure management guidance [7]. SGLT2 inhibitor therapy is typically initiated first, with attention to the expected early eGFR dip reported at roughly 3–6 mL/min/1.73m^2^, which is generally transient and does not warrant discontinuation in the absence of other adverse events [82]; tirzepatide can then be introduced at a low starting dose with gradual up‐titration according to gastrointestinal tolerance, mirroring the titration approach used in the SUMMIT trial [10]. During escalation, diuretic dosing should be reassessed to avoid additive volume depletion and patients should be monitored clinically and biochemically over the following weeks, with dose adjustments guided by weight, renal function and symptom trajectory at routine follow‐up, an approach extrapolated from individual‐agent trial protocols and general HF monitoring principles, since no dedicated trial has yet tested a formal combined titration protocol [7, 10, 65].

## 6 Conclusion

Heart failure with preserved ejection fraction (HFpEF) accounts for over one‐half of heart failure hospitalizations, with rising prevalence linked to aging, hypertension, metabolic syndrome, renal dysfunction and obesity. Obesity contributes to HFpEF through increased epicardial adipose tissue, chronic inflammation and elevated cardiac output leading to ventricular hypertrophy.

Tirzepatide and SGLT2 inhibitors act through distinct but complementary mechanisms in obesity‐related HFpEF. Tirzepatide produces substantial weight loss and broader metabolic improvement, including gains in KCCQ score and reductions in paracardiac fat, while SGLT2 inhibitors provide foundational haemodynamic benefit through natriuresis, decongestion and improved myocardial energetics, independent of diabetes status. Because SUMMIT and EMPEROR‐Preserved were not designed for head‐to‐head comparison, neither agent should be considered superior to the other based on current evidence.

The rationale for combining these two drug classes is physiologically coherent and mechanistic and observational data suggest complementary benefit across the cardio‐renal‐metabolic axis. However, this combination has not been evaluated in a dedicated randomized controlled trial in obesity‐related HFpEF and its long‐term safety, including the risk of additive volume depletion and hypotension, remains unestablished. Combination therapy should therefore be regarded as a promising but unproven strategy, and clinicians who consider it should apply careful individualized monitoring pending confirmatory trial data.

## Author Contributions

**Abdinasir Adam Shidane:** conceptualization, investigation, writing – review and editing, writing – original draft, validation. **Anas Ali Abdi:** conceptualization, investigation, writing – original draft. **Zakariye Isak Mohamed:** conceptualization, writing – original draft, investigation, writing – review and editing, validation. **Mohamed Hassan Hussein:** conceptualization, investigation, writing – original draft. **Abdullahi Mohamud Abdiasis:** conceptualization, writing – original draft, investigation. **Mohamed Abdullahi Mohamud:** conceptualization, writing – review and editing, supervision, validation. **Ali Jimale Mohamed:** conceptualization, writing – review and editing, supervision, validation. **Ayaanle Osman Ibrahim:** conceptualization, investigation, writing – original draft. **Kowthar Ismail Hashi:** conceptualization, investigation, writing – original draft.

## Funding

The authors have nothing to report.

## Ethics Statement

The authors have nothing to report.

## Consent

The authors have nothing to report.

## Conflicts of Interest

The authors declare no conflicts of interest.

## Data Availability

Data sharing not applicable to this article as no datasets were generated or analysed during the current study.
